# A population-based study of self-reported adverse drug events among Lebanese outpatients

**DOI:** 10.1038/s41598-021-87036-x

**Published:** 2021-04-12

**Authors:** Elsy Ramia, Rony M. Zeenny, Souheil Hallit, Pascale Salameh

**Affiliations:** 1 Department of Pharmacy Practice, School of Pharmacy, Lebanese American University, P.O. Box S-23, Byblos, Lebanon; 2 Department of Pharmacy, American University of Beirut-Medical Center, 236, Riad El Solh, P.O.Box11, 1107-2020 Beirut, Lebanon; 3 Faculty of Medicine and Medical Sciences, Holy Spirit University of Kaslik (USEK), Jounieh, Lebanon; 4 Research Department, Psychiatric Hospital of the Cross, Jal Eddib, Lebanon; 5 INSPECT-LB: National Institute of Public Health, Clinical Epidemiology and Toxicology, Beirut, Lebanon; 6 Faculty of Pharmacy, Lebanese University, Beirut, Lebanon; 7 Faculty of Medicine, University of Nicosia, Nicosia, Cyprus

**Keywords:** Health care, Medical research, Risk factors

## Abstract

There is a limited number of studies assessing the epidemiology of Adverse Drug Events (ADEs) in the outpatient setting, especially those that do not result in healthcare use**.** The primary objective of this study was to assess the prevalence and determinants of self-reported ADEs among Lebanese outpatients. It was a cross-sectional observational study performed among Lebanese outpatients visiting community pharmacies across Lebanon. A questionnaire was designed to elicit patients’ relevant information. The association between categorical variables were evaluated using Pearson χ^2^ test or Fisher's exact test. Binary logistic regression was performed to identify factors that affect the experience of self-reported ADEs. The study comprised 3148 patients. Around 37% of patients reported experiencing an ADE in the previous year. When ADEs occur, 70.5% of the respondents reported informing their physicians. Increasing number of medications per patient, use of injectable medication, and inquiring about potential drug-drug interactions were associated with higher experience of ADEs (*p* = 0.049; *p* = 0.003; and *p* = 0.009 respectively). Patients who received hospital discharge counseling reported experiencing less ADEs (*p* = 0.002). Our study showed prevalence of ADEs among Lebanese outpatients especially patients with polypharmacy, and highlighted the need to educate patients about the importance of reporting ADEs to their physicians.

## Introduction

Patient safety is a serious public health concern across the world. According to the World Health Organization (WHO), “there is a 1 in 300 chance of a patient being harmed during health care”^1^. Adverse drug events (ADEs) are the most common type of adverse events experienced by patients^2^. An ADE is “any injury occurring during the patient’s drug therapy and resulting either from appropriate care, or from unsuitable or suboptimal care”. Hence, ADEs include the adverse drug reactions (ADRs) occurring during normal use of the medicine, and any harm secondary to a medication error or inappropriate medication use^3,4^. Although estimates of the incidence of ADEs vary depending on the setting, the population, and the method of assessment used, ADEs remain an important cause of morbidity, mortality and wasted expenditure^5,6^.

There are several reliable methods to collect ADEs. These methods comprise spontaneous reporting through national pharmacovigilance databases, collecting practice data, soliciting events from healthcare professionals, direct observation, and surveying patients for drug-related events^7,8^.

In fact, patients’ role in reporting ADEs is well recognized and the merits of patient reports are considered internationally^9^. The European Medicines Agency (EMA) and the United States Food and Drug Administration (US FDA) have made it possible for patients to report suspected ADEs directly through their national reporting databases^8,10^. Patients are encouraged to act as “vigilant partners” in their own care as they can decrease the risk of drug therapy^9,11^. Despite some limitations including reports from patients complement professionals’ reports, and can trigger major considerations, labelling changes, or drug withdrawal from the market^12,13^.

Similarly, patient surveys are an important and reliable method to detect ADEs in the outpatient setting. Outpatients’ ADEs are often under-reported, poorly documented, and not confirmed by a healthcare professional^14,15^. In contrast to inpatients, outpatients are responsible for both obtaining and administering their medications, and tend to have longer duration of treatment. Furthermore, outpatients often have more than one prescriber, less regular contact with their physicians and far less monitoring compared with hospitalized patients^14,16^.

There is a limited number of studies assessing the epidemiology of ADEs in the outpatient setting, especially the ADEs that do not result in healthcare use^17,18^. Few published studies examined ADEs from the patients’ perspective, and aimed at estimating the prevalence and assessing risk factors of self-reported ADEs across Europe, the United States and Australia^19–23^. Additional studies highlighted poor patient awareness as a main barrier for outpatient reporting of ADRs^24,25^.

In Lebanon, the financial constraints, fragmented care, and easy accessibility of medications, potentially increase the risk of inappropriate medication use and adverse drug events^26^. Cross-sectional studies conducted among Lebanese outpatients found potentially inappropriate medication use^27–29^, incomplete performance of follow-up monitoring^30^, and suboptimal patient knowledge of their prescribed medications’ ADRs^31^. Other studies also highlighted a poor reporting culture among Lebanese medical staff and suboptimal pharmacovigilance awareness^32–35^.

Lastly, Lebanese pharmacists working in community settings reported being aware of ADRs occurring with various medications post-marketing, yet were currently unable to disseminate this information or record it centrally, in the absence of an active national reporting system^36^.

There is a lack of studies addressing outpatients’ experience of ADEs in Lebanon. The primary objective of this study was to assess the prevalence and risk factors of self-reported Adverse Drug Events among Lebanese outpatients. A secondary objective was to assess the counseling outpatients received from their primary caregivers in the community and upon hospital discharge when applicable.

## Methods

A cross-sectional questionnaire-based observational study was performed between March and May 2016, among Lebanese outpatients visiting a community pharmacy. Patients were eligible for the study if they were Lebanese adults, receiving one or more medication, and willing to participate in the study.

### Data collection tool

The investigators derived the questionnaire from the National Health Service-England (NHS) inpatient survey program, the WHO “Patient Questionnaire about Medication Safety”^37,38^, and other studies of self-reported ADEs^14,20,39^. The investigators then adapted the questionnaire to the Lebanese context and added many questions to align with the study objectives. The questionnaire addressed the participants’ sociodemographic characteristics, medical condition, outpatient risk-associated behaviors, experience of ADEs, and hospitalization during the previous year (if any).

Throughout the questionnaire, investigators measured frequency using a five-point Likert scale with answer categories ranging from always to never. The questionnaire was first developed in English, and was then translated into Arabic using a forward–backward translation process for validation. Before administration, the questionnaire was pilot-tested for clarity and content validity in different populations, including health professionals, nonprofessionals, and the elderly.

### Assessing self-reported ADEs

Patients’ experience of ADEs was assessed by asking the patients whether they experienced “any problems or symptoms” after taking their medication(s) within the past year. Since our adopted definition for ADEs includes both ADRs and medication errors, and since the medication administration in the outpatient setting is under the control of the patient, the distinction between an ADR and a medication error is not straightforward and cannot be assessed using a simple self-administered questionnaire. Therefore, the investigators opted to ask about any “symptoms/problems” the patient could have experienced after medication administration, as a general question that includes either an ADR or a potential medication error. The questionnaire also included questions about the outcome of the most serious symptom/problem that patients had, and about reporting these symptoms to their physicians^20^.

### Data collection process

Inspectors affiliated with the Order of Pharmacists of Lebanon (OPL) performed the data collection. The study investigators trained fourteen OPL inspectors, all pharmacists, in a 4-hour session that included a general overview of ADEs, instructions on how to include participants in the study, and a review of the questionnaire.

The investigators obtained a list of all active community pharmacies in Lebanon from the OPL. Using convenience sampling, the investigators selected a sample of 1574 community pharmacies across Lebanon for patient encounters. The OPL inspectors visited each pharmacy for a maximum of one hour. In each pharmacy, one inspector approached patients asking for their willingness to participate, and described the objective of the study and the estimated time to complete the questionnaire. The inspector highlighted that participation in the study is voluntary and strictly confidential. The first two patients visiting the pharmacy during the inspector’s presence and agreeing to participate were included. The questionnaire was self-administered.

### Ethical approval and consent to participate

The study was performed in accordance with the ethical standards as laid down in the 1964 Declaration of Helsinki and its later amendments. Before initiation of the data collection, the approval of the Lebanese American University Institutional Review Board (LAU IRB) was sought as appropriate. IRB registration number: #IRB00006954 LAUIRB#1. Accordingly, all participants provided informed oral consent prior to participation.

### Data management and statistical analysis

No quantitative data exists for the experience of ADEs in the Lebanese population. The investigators based their sample size estimation on a study performed among 7099 Swedish adults, where ADEs were reported by 19.4% of the respondents^20^. Estimating the Lebanese population size to be around 5,000,000^40^, we defined our goal to interview a minimum sample size of 920 patients from 460 community pharmacies. This sample size is powered to provide 95% confidence level.

The data was analyzed using SPSS version 23 software. Descriptive statistics were used to calculate and report all participants’ responses. For all the analysis, the five-point Likert scale was dichotomized into Yes (always/very often/sometimes) and No (rarely/never). The association between categorical variables were evaluated using Pearson χ^2^ test or Fisher’s exact test where the expected cell count was less than 5. Binary logistic regression was performed to identify factors that were associated with the self-reported experience of ADEs (dichotomized), using a Backward LR method. Variables with a *p*-value of 0.2 or less in the bivariate analysis were included in the final models. A *p*-value of less than 0.05 was considered significant.

## Results

The study comprised 3148 patients from different geographical areas around Lebanon. The study acceptance rate was 78%. Patients denied participation due to lack of time or no interest.

Study participants had almost equal gender distribution and had a mean age of 54.33 years (SD 16.03). When examining patients’ medical conditions, around 20% of the study participants reported having  3 or more concomitant chronic diseases, and around 14% taking  5 or more medications/day (Table 1).

**Table 1:** Sociodemographic characteristics and medical condition.

Characteristic	Frequency (%)^a^
**Gender**	
Male	1550 (49.2)
Female	1559 (49.5)
**Age (mean in years ± SD)**	54.33 ± 16.03
**Marital status**	
Single	464 (14.7)
Married	2350 (74.7)
Widowed	261 (8.3)
Divorced	42 (1.3)
**Level of education**	
Illiterate	187 (5.9)
Some school	936 (29.7)
High school	1156 (36.8)
Bachelor degree	488 (15.5)
Master’s degree	232 (7.4)
Doctoral degree	70 (2.2)
**Employment status**	
Student	61 (1.9)
Self-employed	686 (21.8)
Employed	802 (25.5)
Unemployed	983 (31.2)
Retired	455 (14.5)
**Geographic area of residence**	
Beirut	523 (16.6)
Mount Lebanon	1486 (47.2)
North	180 (5.7)
Bekaa	495 (15.7)
South/Nabatiyye	438 (13.9)
**Number of Chronic Diseases per patient**	
1	1693 (53.8)
2	714 (22.7)
≥ 3	635 (20.2)
**Number of medications taken at home every day**	
1	371 (11.8)
2	598 (19.0)
3	981 (31.2)
4	615 (19.5)
≥ 5	432 (13.7)
**Chronic diseases (most common)**	
Hypertension	1399 (44.4)
Dyslipidemia	992 (31.5)
Diabetes Mellitus	840 (26.7)
Rheumatoid Arthritis	280 (8.9)
Asthma/COPD	237 (7.5)
Chronic Heart Failure	144 (4.6)
Depression	50 (1.6)
Osteoporosis	41 (1.3)
Intake of any oral liquid medication	170 (5.4)
Intake of any inhaled medication	209 (6.6)
Intake of any patch medication	24 (0.8)
Intake of any injectable medication	227 (7.2)

^a^Sometimes the cumulative percentages may not reach 100% due to missing values. When missing values are less than 10%, they were not reported explicitly.

### Self-reported ADEs

Around 37% of our patient population reported experiencing an ADE. While 18.7% of the ADEs were mild requiring no change in therapy, the most serious ADEs experienced by the patient resulted in dose reduction (18.1%), change of therapy (42.1%), hospitalization (5.1%) or long-term complications (1.5%). When ADEs occur, 70.55% of the respondents reported informing their physicians. Among the 344 patients who reported not informing their physicians about their ADEs, around 17% reported being unable to reach their physicians to report ADEs, 16% were not educated to report ADRs as they occur, and 14.8% reported that their physicians are not usually welcoming to discuss their concerns (Table 2).

**Table 2:** Self-reported experience of Adverse Drug Events (ADEs).

Outcome	Frequency (%)^a^
**Patients’ self-reported experience of ADEs**	
Yes	1168 (37.1)
No	1980 (62.9)
**Main outcome of most serious ADE** (for those who answered yes on question 1)	
Mild reaction, No change in therapy	219 (18.7)
Reaction required dose reduction	211 (18.1)
Reaction required change of therapy	492 (42.1)
Reaction required treatment	44 (3.8)
Reaction required hospitalization	59 (5.1)
Reaction resulted in a long term complication	18 (1.5)
Missing answer	125 (10.7)
**Informing the physician when ADEs occur** (for those who answered yes on question 1)	
Yes	824 (70.55)
No	344 (29.45)
**Reason for not informing the physician about ADEs** (for those who answered No on question 2)	
Mild reaction, no need to inform physician	167 (48.55)
Unable to reach the physician	59 (17.15)
Physician did not inform patient to report in case of ADR	55 (15.99)
Physician not usually welcoming to discuss patient’s concerns	51 (14.82)

^a^Sometimes the cumulative percentages may not reach 100% due to missing values. When missing values are less than 10%, they were not reported explicitly.

In the multivariable analysis, several variables were significantly associated with patients’ reported experience of ADEs. Increasing number of medications taken at home every day was significantly associated with higher experience of ADEs (ORa = 1.225, *p* = 0.049). Patients who reported using an injectable medication (ORa = 3.008, *p* = 0.003) and those who asked their pharmacists about the possible interactions with prescribed medications while getting the OTC (Over-the-counter) medications from the pharmacy (ORa = 1.945, *p* = 0.009) reported experiencing more ADEs. Patients who received counseling from their physicians regarding missing drug doses, and for whom a member of hospital staff explained how to take the medications in an understandable way before hospital discharge reported experiencing less ADEs (ORa = 0.439, *p* = 0.004; and ORa = 0.430, *p* = 0.002 respectively). The variables “physician assessing medication history before prescribing a new medication” and “physician inquiring about previous ADRS before prescribing a new medication” remained in the final model with a non-significant p-value of 0.075 and 0.088 respectively. Having any of the chronic disease did not significantly affect the experience of ADEs (Table 3).

**Table 3:** Patients’ self-reported experience of Adverse Drug Events (ADEs)—Multivariable Analysis.

Variable^a^	ORa	Confidence interval	*P*-value
**Geographic area of residence (Beirut is the reference)**			
Mount Lebanon	0.498	0.262–0.948	0.034
North	2.840	0.502–16.071	0.238
Bekaa	1.105	0.508–2.399	0.802
South/Nabatiyye	0.378	0.091–1.571	0.181
Number of medications taken at home every day	1.225	1.001–1.499	0.049
Intake of any injectable medication	3.008	1.465–6.177	0.003
Asking about possible interactions with prescribed medications while getting over-the-counter medications from the pharmacy	1.945	1.183–3.199	0.009
Physician providing counseling about missing doses	0.439	0.251–0.768	0.004
Physician assessing medication history before prescribing a new medication	1.730	0.947–3.162	0.075
Physician inquiring about previous ADRs before prescribing a new medication	1.599	0.932–2.742	0.088
A member of hospital staff explaining to the patient how to take the medications in an understandable way before hospital discharge	0.430	0.252–0.733	0.002

^a^Variables with a *p*-value of 0.2 or less in the bivariate analysis were included in the initial model. Those include: gender; marital status; level of education; geographic area of residence; employment status; number of chronic diseases per patient; chronic kidney disease; intake of any inhaled medication; intake of any injectable medication; initiation of medication-related discussion; not taking a prescribed medication because of counter-indications mentioned in the leaflet and not mentioned by the physician during the visit; asking about possible interactions with prescribed medications while getting the OTC’s from the pharmacy; pharmacist providing counseling about drug interactions, missing doses, accidental overdose, and potential ADRs; physician providing counseling about drug interactions, missing doses, accidental overdose, and potential ADRs; physician assessing medication history before prescribing a new medication; physician inquiring about previous ADRs before prescribing a new medication; hospitalization in the previous year; a member of hospital staff explaining to the patient the purpose of the medications to be taken at home in an understandable way; a member of hospital staff explaining to the patient how to take the medications in an understandable way. Categorical variables identified: level of education, geographic area of residence, employment status, initiation of medication-related discussion.

Using a Backward LR method, the model finally retained the variables shown in this table. Hosmer and Lemshow test for sample adequacy *p*-value: 0.451. Nagelkerke model summary 0.223.

### Other outcomes

#### Outpatients’ medication-related practices

The study participants reported several suboptimal medication-related practices. Around 12% of the study population reported acquiring their medications from different community pharmacies, and around 80% reported not considering counseling services when selecting their community pharmacy of choice. In terms of medication administration, the majority of participants reported using non-calibrated measures for intake of liquid medications (81% for teaspoon and tablespoon); and around 12% reported having a neighbor/relative administer injectable medications. At the level of drug information, around 36% reported not discussing the medications they take each time they visit the physicians, around 61% reported not reading the leaflet of each medication they take, and around half of the patients didn’t ask about the possible interactions between the over-the-counter drugs and the medications they take.

Around 88% of the patients reported not taking a prescribed medication because of contraindications mentioned in the leaflet and not mentioned by the physician during the visit (Table 4).

**Table 4:** Outpatients’ medication-related practices.

Outcome	Frequency (%)^a^
**Source of medication acquisition**	
Same pharmacy	2658 (84.4)
Different pharmacies	375 (11.9)
Other	51 (1.6)
**Preference for pharmacy selection (check all that apply)**	
Trust the pharmacist	2056 (65.3)
Insurance selection	72 (2.3)
Proximity to house/work	1458 (46.3)
Easy access and parking	242 (7.7)
Discount	106 (3.4)
Counseling	648 (20.6)
**Tools used to measure liquid dose of medication**	
Teaspoon	21 (12.5)
Tablespoon	115 (68.5)
Calibrated cup/syringe	25 (14.9)
**Administration of injectable medication performed by**	
Self	90 (41.1)
Neighbor/relative	26 (11.9)
Pharmacist	69 (31.6)
Physician/nurse at home	24 (11.0)
Healthcare provider in outpatient clinics	10 (4.6)
**Discussion of prescribed medications with physician during visit/consultation**	
Yes	1915 (60.8)
No	1145 (36.4)
**Initiation of medication-related discussion**	
Physician	1104 (35.1)
Patient	1636 (52.0)
Accompanying person	290 (9.2)
**Reading the leaflet of each medication**	
Yes	1162 (36.9)
No	1911 (60.7)
**Not taking a prescribed medication because of counter-indications mentioned in the leaflet and not mentioned by the physician during the visit**	
Yes	278 (8.8)
No	2772 (88.1)
**Asking about possible interactions with prescribed medications while getting the OTC’s from the pharmacy**	
Yes	1420 (45.1)
No	1648 (52.4)

^a^Sometimes the cumulative percentages may not reach 100% due to missing values. When missing values are less than 10%, they were not reported explicitly.

#### Counseling provided by healthcare providers regarding elements of medication use

Significantly more patients reported receiving counseling from pharmacists as compared to physicians regarding elements of medication use: drug interactions (78.4% vs 34.3%, *p* < 0.001); missing drug doses (55.7% vs 31.4%, *p* < 0.001); accidental overdose (49.9% vs 28.7%, *p* < 0.001); and potential ADRs (67.1% vs 38.8%; *p* < 0.001). While 73.5% of our patient population reported that physicians assessed their medication history before prescribing a new drug, only 49.7% reported physicians inquiring about previous ADRs before prescribing a new medication (Table 5).

**Table 5:** Counseling provided by healthcare providers regarding elements of medication use.

Outcome	Physician n (%)^a^	Pharmacist n (%)^a^	*P*-value
Providing counseling about drug interactions	1080 (34.3)	2468 (78.4)	< 0.001
Providing counseling about what to when missing a drug dose	988 (31.4)	1754 (55.7)	< 0.001
Providing counseling about accidental overdose	904 (28.7)	1571 (49.9)	< 0.001
Providing counseling about potential ADRs	1221 (38.8)	2112 (67.1)	< 0.001
Assessing medication history before prescribing a new medication	2315 (73.5)		
Inquiring about previous ADRs before prescribing a new medication	1564 (49.7)		

^a^Sometimes the cumulative percentages may not reach 100% due to missing values. When missing values are less than 10%, they were not reported explicitly.

#### Hospitalization in the previous year

Around 23% of our patient population reported hospitalization in the previous year, with a mean length of stay of 5.2 days (± 2.8 days). The majority of hospitalized patients (85%) reported being prescribed medications upon discharge, with 71.6% of them being informed by a healthcare provider about the purpose of the medications and how to take it. Only 27.8% of patients reported being counseled about ADRs of the prescribed medications, and 56.1% being given written or printed medication information upon discharge (Table 6).

**Table 6:** Hospitalization in the previous year.

Outcome	Frequency (%)^a^
**Hospitalization in the previous year**	
No	2335 (74.2)
Yes	710 (22.6)
**Length of hospital stay**	
Mean	5.18 days
Minimum	1 day
Maximum	17 days
SD	2.84
**Admission to a critical care area (ICU/CCU/CSU)**^**b**^	
No	563 (79.5)
Yes	145 (20.5)
**Patient prescribed medications upon discharge**^**b**^	
No	105 (14.8)
Yes	605 (85.2)
**A member of staff explaining to the patient the purpose of the medications to be taken at home in an understandable way**^**c**^	
No	172 (28.4)
Yes	433 (71.6)
**A member of staff explaining to the patient how to take the medications in an understandable way**^**c**^	
No	172 (28.4)
Yes	433 (71.6)
**A member of staff explaining to the patient about ADRs to watch for**^**c**^	
No	436 (72.2)
Yes	168 (27.8)
**Given written or printed information about medications upon discharge**^**c**^	
No	265 (43.9)
Yes	338 (56.1)

^a^Sometimes the cumulative percentages may not reach 100% due to missing values. When missing values are less than 10%, they were not reported explicitly.

^b^Total number of participants on the marked questions is 710, referring to the patients who were hospitalized during the last year.

^c^Total number of participants on the marked questions is 605, referring to the patients who were prescribed medications upon hospital discharge.

## Discussion

In this cross-sectional questionnaire-based study, around 37% of our patient population reported experiencing an ADE within the previous year. When ADEs occur, 70.5% of the respondents reported informing their physicians.

As previously mentioned, there are several reliable methods to collect patient ADEs. Different methods of collecting patient data regarding adverse events (i.e. patient surveys versus spontaneous reporting) lead to large differences in the reported rates of these adverse events, reducing the validity and meaningfulness of comparisons^41^. In this discussion, the authors opted to compare the results with findings of other patient survey studies.

The rate of ADEs reported by our study participants was similar to the findings of a recently published questionnaire-guided study of 1190 ambulatory adult patients in Nigeria^42^, but higher than previously published rates of self-reported ADEs^20–22^. In fact, the percentage of self-reported ADEs in questionnaire-based studies depends on the definition of ADEs adopted and the timespan specified for the reported ADEs^20–22^. In a study by Oladimeji et al. assessing self-reported ADEs among elderly US residents enrolled in Medicare, 18% of the respondents reported an ADE in the past year^22^. In that study, authors defined an ADE as the patient visiting a physician to report an unwanted reaction or medicine problem^22^. This can explain the higher percentage reported in our study, since we inquired about any ADE, even if it did not lead to a physician’s visit. In a study by Hakkarainen et al. that aimed to assess the 1-month prevalence of self-reported ADEs among the adult public in Sweden, 19.4% of the respondents reported experience of ADEs^20^. This lower percentage can be explained by the 1-month timespan specified. In another population based-study in Sweden by Hedna et al., the authors reasoned that the low reported ADRs percentages (2.5%) could reflect a lack of patient awareness for symptoms of ADRs^43^. In fact, many ADEs are likely never reported because they are not recognized^8^. Lastly, in a national cross-sectional study assessing community-based ADRs in Saudi Arabia, the sample prevalence of ADRs was around 23%. Authors, however, did not assess ADEs in that survey which does not compare with our study^44^.

Early identification of ADEs and factors associated with them may help physicians prevent and resolve these ADEs^45^. Around 70% of our participants reported informing their physicians about their experienced ADEs. This percentage falls within the range reported in published literature where proportions of patients who claimed informing their physicians about their ADRs ranged between 54 and 87% of respondents^46,47^. Reasons that our participants stated for not informing their physicians include their inability to reach the physician or them not being adequately instructed to report. Physicians hold a key responsibility in educating patients about ADEs and the importance of reporting ADEs as they occur, as well as in facilitating the communication and the reporting mechanism. A study examining barriers and facilitators of adverse event reporting by adolescent patients and their families showed that the quality of healthcare experience and the type of communication with the healthcare provider influenced patient reporting^48^.

In our findings, the occurrence of ADEs was positively associated with the number of daily medications taken, and the use of an injectable medication. The association between the number of medications taken and the self-reported ADEs is documented in the literature^19^. There is no evidence that intake of injectable medications is associated with higher ADRs, but research shows that the incidence of errors with injectable medications is higher than with other forms of medications^49^. Patients who reported experiencing ADEs also reported asking about possible interactions with prescribed medications while getting the OTC’s from the pharmacy. This association can be explained by the fact that patients who experience more ADEs may become more concerned about potential drug interactions. In addition, patients who received counseling from their physicians regarding missing drug doses, and who received medication counseling before hospital discharge reported experiencing less ADEs. In fact, studies have shown an association between discharge counseling and lower rates of adverse drug events and hospital re-admission^50,51^.

Our findings show that significantly more outpatients reported receiving counseling from pharmacists as compared to physicians regarding elements of medication use. In fact, physicians commonly fail to provide appropriate counseling about prescription medications^52–55^. One of the main reported physicians’ expectations from pharmacists in primary care was to provide more education and counseling about medications^56,57^. While pharmacists have a professional responsibility to provide patient counseling, physicians can assume a bigger role in providing medication counseling.

To our knowledge, this is the first study in the Lebanese population, on a national scale, that addresses self-reported experience of ADEs. In fact, there are differences among countries in the occurrence of ADRs due to many differences including diseases, prescribing patterns, genetics, and drug distribution. Data derived from within the country may have greater relevance and educational value and may encourage national regulatory decision-making^58^.

### Study limitations

Our study population consisted of outpatients visiting the community pharmacies to procure their medications, which excludes patients who acquire their medications from dispensaries, and patients with lower healthcare accessibility. There is also potential information bias. The patients’ experience of ADEs assessed in our study was self-reported, and was not verified or confirmed by our investigators using objective evidence.

At the time investigators conducted this study, there was no active national pharmacovigilance center in Lebanon, through which healthcare professionals or patients can report ADEs. ADEs were not reported, analyzed, or registered centrally. Following the completion of this study, the medication safety subcommittee of the OPL created an electronic platform for pharmacists to report ADRs^59^. This platform remains inactive until date due to poor reporting. Moreover, the Lebanese University—School of pharmacy had established in 2004 a center for Adverse Events of Drug Monitoring that remained inactive until 2020. A collaborative agreement between the Lebanese University and the Ministry of Public Health (MOPH) reactivated the center (ministerial decree 427/1) and authorized it to function officially as the national pharmacovigilance center. Stakeholders have just completed all the preparations and documentations needed to launch the center for service in 2021 and become a full member in the Uppsala network^60^.

## Conclusions

Our study showed prevalence of ADEs among Lebanese outpatients especially patients with polypharmacy, and suggested that patients do not report all the ADEs to their physicians. Patients should be educated on the importance of reporting ADEs to their primary healthcare provider. Reporting helps preventing future ADEs, contributes to the public knowledge and medical literature, and encourages national regulatory decision-making. There is need to assess potential preventability of these ADEs through proper follow-up and monitoring, in an attempt to reduce potential patient harm and healthcare costs. Studies assessing Lebanese inpatients experience of ADEs can complement this data throughout the continuum of healthcare, and can drive evidence-based decision-making.

## Data Availability

The datasets used and/or analyzed during the current study are available from the corresponding author on reasonable request.
